# Using a brain-like endothelial cell differentiation to characterize the CS79iBRCA-n2 BRCA1 mutated patient derived stem cell line

**DOI:** 10.3389/fcell.2025.1516669

**Published:** 2025-04-30

**Authors:** Natalie G. Alexander, Kylie A. Buchanan, Alexandra E. Meyer, Lauren M. Mitterway, Caroline O. Vanderburgh, Shreyas S. Rao, Brandon J. Kim

**Affiliations:** ^1^ Department of Biological Sciences, University of Alabama, Tuscaloosa, AL, United States; ^2^ Department of Chemical and Biological Engineering, University of Alabama, Tuscaloosa, AL, United States; ^3^ Center for Convergent Biosciences and Medicine, University of Alabama, Tuscaloosa, AL, United States; ^4^ Alabama Life Research Institute, University of Alabama, Tuscaloosa, AL, United States; ^5^ Department of Microbiology, Heersink School of Medicine, University of Alabama at Birmingham, Birmingham, AL, United States; ^6^ Department of Biological Sciences, University of Texas at Dallas, Richardson, TX, United States

**Keywords:** blood-brain barrier, patient derived stem cells, *BRCA1* mutation, brain endothelial cells, induced pluripotent stem cells, disease modeling

## Abstract

*BRCA*1/2 genes are considered tumor suppressor genes and help repair damaged DNA. Pathogenic germline mutations of *BRCA1/2* genes are the most common hereditary cause of breast cancer and ovarian cancer. It has been established that *BRCA1* mutations increase the risk of brain metastasis compared to the *BRCA1* wildtype, and once metastasis occurs to the brain the disease is considered uncurable. The blood-brain barrier (BBB) is essential for maintaining and regulating homeostasis of the central nervous system and is composed of highly specialized brain endothelial cells. Using a human induced pluripotent stem cell (hiPSC) based model, we characterized an hiPSC line from an invasive cancer patient harboring a *BRCA1* mutation. This patient-derived hiPSC line can be utilized to study BBB properties as after differentiation into brain-like endothelial cells (BECs), BECs derived from this line express BBB markers such as tight junction proteins, and functional efflux transporters. Future application of patient-derived stem cell models could provide a platform to discover genetic predispositions to BBB disruption in individuals with *BRCA1* mutations, as well as the potential molecular mechanisms contributing to brain metastasis.

## Introduction

In the United States, cancer continues to be the second-leading cause of death after cardiovascular disease (Siegel et al., 2024; Wang et al., 2016; Nagai and Kim, 2017). BReast CAncer (*BRCA*) genes, including *BRCA1* and *BRCA2*, are tumor suppressor genes, whose role is established to repair damaged DNA (Boulton, 2006; Mai et al., 2009). Consequently, mutations in *BRCA1/2* genes hinder proper DNA repair and have been shown to increase the malignancy risk for hereditary cancers, especially breast and ovarian cancers (Gudmundsdottir and Ashworth, 2006; Ratner et al., 2019). *BRCA1* mutations have also been associated with earlier diagnosis and progression of brain metastases (Ratner et al., 2019). In ovarian cancer patients, the *BRCA1* mutation has shown a 4-fold greater risk for brain metastases compared to the *BRCA1* wildtype and has led to an 8-month earlier diagnosis of brain metastasis (Ratner et al., 2019). Similarly, for central nervous system metastatic breast cancer, *BRCA1/2* mutations have shown a younger median age for diagnosis, a greater incidence of high-grade tumors, and a lower survival time post-diagnosis than wild-type *BRCA1* individuals (Ben-Zion Berliner et al., 2024). To metastasize to the brain, cancer cells in circulation must interact with and penetrate the blood-brain barrier (BBB) (Arshad et al., 2011).

The BBB is comprised of highly specialized brain endothelial cells (BECs) that encompass the cerebral vasculature (Abbott et al., 2010; Knopp et al., 2022). The BBB is essential for the maintenance and regulation of the neural microenvironment and prevents the entry of pathogens and toxins into the CNS (Helms et al., 2016; Patabendige and Janigro, 2023). BECs serve as the interface between the circulation and the CNS and express complex tight junctions (Rubin and Staddon, 1999). Due to the presence of efflux transporters and low endocytosis rates, the BBB restricts 100% of large molecule drugs and 98% of small molecule drugs which poses an obstacle for drug delivery of chemotherapeutics (Pardridge, 2007; Hersh et al., 2016).

BBB models present unique challenges as robust *in vivo* modeling may have interspecies variation, and *in vitro* modeling of primary cells are limited by passage number and often lose BBB-defining phenotypes when removed from the neural microenvironment (Helms et al., 2016; Obermeier et al., 2013; Andjelkovic et al., 2020; He et al., 2014). Human stem cell (hSC) technology has allowed for the advancement of *in vitro* modeling and overcomes challenges such as the loss of BBB-defining phenotypes, scalability, and interspecies variations (Obermeier et al., 2013; Verscheijden et al., 2021; Wilhelm and Krizbai, 2014).

Previous studies have successfully used patient derived stem cells to model specific disease contexts in the BBB (Vatine et al., 2017; Yucer et al., 2021; Ozgür et al., 2023). Patient derived hiPSCs harboring MCT8 mutations have been used to model the BBB and study drug transport in a diseased BBB (Vatine et al., 2017).

It has also been demonstrated that hiPSC-BECs derived from Huntington’s disease patients had key transcriptional and functional differences compared to healthy control hiPSC-BECs, indicating a crucial deficit in BBB function in this neurodegenerative disease context (Lim et al., 2017). *BRCA1* mutated cells lines have been used to model fallopian tube epithelium and recapitulated ovarian carcinogenesis (Yucer et al., 2021). Patient derived models provide the ability to model human diseases with a specific genetic predisposition (Vatine et al., 2017). However, to our knowledge, hiPSC-BEC models derived from *BRCA1* mutated patients have not been reported.

In this study, we use an induced pluripotent stem cell model, where over the period of 13 days, patient-derived hiPSCs are differentiated, expanded, and purified with the result of patient-derived brain-like endothelial cells (Lippmann et al., 2012; Espinal et al., 2022; Lippmann et al., 2014). Recent work has demonstrated that while BEC differentiation is robust, some human iPSC lines may differ in the BBB properties exhibited after differentiation (Vatine et al., 2017; Ozgür et al., 2023; Lim et al., 2017; Patel et al., 2017). For example, the SBAD0201 stem cell line showed non-functional efflux transporter P-glycoprotein (P-gp) (Ozgür et al., 2023). Throughout the study we use the well-characterized hiPSC line IMR90-4 for comparison of key BBB marker expression after differentiation. The goal of this study was to characterize the BEC differentiation and resulting BBB-like properties of the *BRCA1* mutated patient-derived stem cell line CS79iBRCA-n2 *in vitro*.

## Methods

### Cell lines

The CS79iBRCA-n2 cell line was purchased from Cedars-Sinai Biomanufacturing Center. Lymphoblastic cells were first harvested from a female early-onset, stage IIIC ovarian cancer patient with a *BRCA1* mutation (IVS5+1G>A, located at the junction between exon five and intron 6). The CS79iBRCA-n2 cell line was reprogrammed from the Cedars-Sinai Biomanufacturing Center parent cell line 2012-0872 4079. Lymphoblastic cells were reprogrammed to iPSCs via an engineered episomal plasmid containing reprogramming factors Oct3/4, Sox2, KLF4, L-Myc, shp53, and Lin28. The IMR90-4 cell line was purchased from WiCell (WISCi004-B) where it was reprogrammed using viral transfection methods from a female parent cell line, IMR90.

### Culturing of hiPSCs

hiPSCs were cultured on Matrigel (Corning; 354234) coated 6-well plates and maintained in StemFlex Medium with supplement (Gibco; A3349401), changed daily (Lippmann et al., 2012; Espinal et al., 2022; Lippmann et al., 2014; Stebbins et al., 2016; Gomes et al., 2019). When iPSCs reach 70% confluence, cells were dissociated from the well using Versene (Gibco; 15040066) and passed at 1:3, 1:6, and 1:12 split ratios onto Matrigel coated 6-well plates. Cells were continuously maintained in StemFlex medium, changed daily. Passages 15–25 were used for differentiations.

### Differentiation of hiPSCs into brain-like endothelial cells

When iPSCs reach 80% confluence, cells were differentiated according to the protocol we and others have used previously (Lippmann et al., 2012; Espinal et al., 2022; Stebbins et al., 2016). Cells were seeded onto Matrigel-coated T75 flasks (VWR; 10861-646) at 10,000 cell/cm^2^ and maintained in StemFlex Medium with supplement for 3 days, with media changed daily. Differentiation into induced brain endothelial cells (BECs) was initiated after 3 days with the addition of unconditioned medium (UM) for 6 days, changed daily (Stebbins et al., 2016). UM media is composed of 89% DMEM/F12 (ThermoFisher; 11330-032), 10% Knockout serum replacement (KOSR) (Gibco; 10828028), 1% Non-essential amino acids (NEAA) (ThermoFisher; 11140-050), 0.5% Glutamax (ThermoFisher; 35050061), and 0.005% β-mercaptoethanol (β-ME). Differentiation was continued with the use of endothelial cell (EC) medium, containing hESFM (ThermoFisher; 11111-044) supplemented with 1% B27 (Gibco; 17504044), 10 µM retinoic acid (RA) (Sigma; R2625), and 20 ng/mL basic fibroblast growth factor (bFGF) (PeproTech; 100-18B-250UG), for 2 days, with media changed daily.

On Day 8, cells are dissociated and singularized using Accutase (StemCell Technologies; MSPP-07920). BECs were then purified by seeding onto ECM coated plates which selectively isolates cells expressing BEC phenotypes and not neural cells (Sigma; C5533-5MG, F1141-5MG). Cells were replated at a seeding density of 500,000 cells/well for 24-well plates and 1 million cells/well for 12-transwell plates in EC + RA medium. Media was changed to EC medium without bFGF and RA supplements (EC - -) 1 day later. BECs were validated through measuring trans-endothelial electrical resistance (TEER) on Day 9 and Day 10, through immunostaining for BEC markers on Day 10, and through functional assays on Day 10.

### P-gp functional assay

Using previously published protocols, P-glycoprotein (P-gp) function in hiPSCs and BECs was determined through the measurement of the accumulation of the P-gp substrate, Rhodamine 123 (R123) (Lippmann et al., 2012; Stebbins et al., 2016; Kim et al., 2019). The potent P-gp inhibitor, Valspodar (Sigma; SML0572-5MG), was utilized to assess P-gp activity for comparison. The cells were washed once with warmed Hank’s Buffer Saline Solution (HBSS) (ThermoFisher; 14065056) and pretreated with or without 10 µM of the inhibitor, Valspodar, with HBSS for 1 h at 37°C and 5% CO_2_. After pretreatment, the cells were then incubated with 10 µM of R123 with or without 10 µM of Valspodar in HBSS for 2 h in 37°C and 5% CO_2_. After incubation, the cells were washed with cold PBS twice and 200 µL of Radioimmunoprecipitation assay (RIPA) buffer was applied. After RIPA buffer application, the plate was then placed on a rotator to shake for 10 min at room temperature while protected from light. The plate fluorescence was then quantified on a plate reader (Molecular Devices SpectramaxiD3). BCA assays (ThermoFisher; 23227) were used to normalize the fluorescent values to account for the number of cells present.

### BCRP functional assay

Breast Cancer Resistance Protein (BCRP) activity in hiPSCs and BECs was measured through the accumulation of the fluorescent BCRP substrate, Hoechst 33342 (ThermoFisher; 62249) (Lippmann et al., 2012; Stebbins et al., 2016). BCRP inhibitor, Ko143 (Enzo: 89158-270), was used as comparison (Kim et al., 2019; Allen et al., 2002; Paturi et al., 2010). The cells were washed once with warm Hank’s Buffer Saline Solution (HBSS) and pretreated with or without Ko143 at 1 µM concentration in HBSS for 1 h at 37°C and 5% CO_2_. After pretreatment, the cells were then incubated with 10 µM of Hoechst 33342 in HBSS with or without inhibitor for 2 h at 37°C and 5% CO_2_. The cells are then washed twice with 500 µL of cold PBS, and then 200 µL of RIPA buffer was applied. The cells were then placed on a rotator for 10 min protected from light. Fluorescence was measured on a plate reader (Molecular Devices SpectramaxiD3). To normalize the fluorescent values, BCA assays (ThermoFisher; 23227) were performed, and OD values were used to normalize.

### RNA isolation and quantitative PCR

RNA was isolated using the NuceloSpin RNA kit (Macherey-Nagel; 740955.50) and cDNA was prepared using LunaScript RT (New England BioLabs; E3010). SYBR green qPCR was run for human *POU5F1* (Oct4) forward primer 5′–CCCCAGGGCCCCATTTTGGTACC – 3′ and reverse primer 5’ – ACCTCAGTTTGAATGCATGGGAGAGC – 3’. 18S rRNA was used as a reference gene for normalization, forward primer 5′–GTAACCCGTTGAACCCCATT – 3′ and reverse primer 5′–CCATCCAATCGGTAGTAGCG – 3′. qPCR was run on a QuantStudio 3 Real-Time PCR system (ThermoFisher). Relative gene expression was calculated using the ΔΔCt method.

### Immunostaining

Both CS79-derived and IMR90-4 derived BECs were immunostained following day 10 of differentiation (Stebbins et al., 2016). CS79-derived hiPSCs were immunostained at 3 days post passaging. Cells were fixed depending on the antibody with either ice cold methanol or 4% paraformaldehyde (diluted in PBS) for 15 min and blocked in 10% Fetal Bovine Serum (FBS) (Table 1) (Stebbins et al., 2016). Markers for immunostaining include Claudin-5, Occludin, ZO-1, P-gp, GLUT1, VE-Cadherin, PECAM-1, and BCRP. Expression was visualized using a Nikon Ti2 inverted epifluorescence microscope equipped with a Qi2 camera (Nikon, Tokyo, Japan) using NiS Elements software version AR.5.30.05 for acquisition. Images were analyzed using ImageJ Software (FIJI).

**TABLE 1:** Antibody table.

Antibody	Species	Company	Catalog #	Dilution	Fixative agent	Secondary	Application
PECAM-1	Mouse	Invitrogen	MA5-13188	1:25	MeOH	Goat anti-mouse IgG (H + L) Alexa Fluor 488 conjugate (A-11001)	Immunostaining
VE-Cadherin	Mouse	Santa Cruz biotechnology	sc-52751	1:25	MeOH	Goat anti-mouse IgG (H + L) Alexa Fluor 488 conjugate (A-11001)	Immunostaining
Claudin-5	Mouse	Invitrogen	35–2500	1:50	MeOH	Goat anti-mouse IgG (H + L) Alexa Fluor 488 conjugate (A-11001)	Immunostaining
ZO-1	Mouse	Invitrogen	33–9100	1:100	MeOH	Goat anti-mouse IgG (H + L) Alexa Fluor 488 conjugate (A-11001)	Immunostaining
GLUT1	Mouse	Invitrogen	MA5-11315	1:200	MeOH	Goat anti-mouse IgG (H + L) Alexa Fluor 488 conjugate (A-11001)	Immunostaining
Occludin	Mouse	Invitrogen	33–1500	1:200	MeOH	Goat anti-mouse IgG (H + L) Alexa Fluor 488 conjugate (A-11001)	Immunostaining
BCRP	Mouse	Millipore sigma	MAB4155	1:50	4% PFA	Goat anti-mouse IgG (H + L) Alexa Fluor 488 conjugate (A-11001)	Immunostaining
P-gp	Rabbit	Invitrogen	MA5-13854	1:25	MeOH	Goat anti-rabbit IgG (H + L) Alexa Fluor 488 conjugate (A-11034)	Immunostaining
VE-Cadherin	Mouse	Santa Cruz biotechnology	sc-52751	1:1000			Western blotting
Claudin-5	Mouse	Invitrogen	352,588	1:1000			Western blotting
ZO-1	Mouse	Thermo fisher	339,100	1:1000			Western blotting
GLUT1	Mouse	Invitrogen	MA5-11315	1:1000			Western blotting
Occludin	Mouse	Invitrogen	33–1500	1:1000			Western blotting
BCRP	Mouse	Millipore sigma	MAB4155	1:500			Western blotting
P-gp	Rabbit	Thermo fisher	PA5-28801	1:000			Western blotting

### Western blot

The protein amounts were derived from BCA assays (ThermoFisher; 23227). The protein samples were heated for 5 min at 95°C before being loaded onto Bolt 4%–12% Bis-Tris Plus protein gels (ThermoFisher; NW04120BOX, NW04125BOX) along with Peacock Plus Prestained Protein Marker (Biotium; 21531) and transferred to nitrocellulose membranes. In the case of BCRP and GLUT1 targeting, 10% β-Mercaptoethanol (β-ME) instead of heat was added to the protein samples. Following 1 h blocking at room temperature with tri-buffered saline +0.1% Tween 20 (1x TBST) and 5% nonfat dry milk, the respective primary antibodies were used for targeting and remained on the membrane overnight at 4°C (Table 1). The next day the blots were washed in TBST (3x) and given goat anti-mouse IgG (H + L) Alexa Fluor 488 conjugate secondary antibody (ThermoFisher; A-11001) for 1 h at room temperature while shaking. For P-gp, goat anti-rabbit IgG (H + L) Alexa Fluor 488 conjugate second antibody (ThermoFisher; A-11034) was used instead. After washing in TBST (3x), the blots were imaged on an iBright FL1500 Imaging System instrument (ThermoFisher) with SuperSignal West Pico PLUS Chemiluminescent Substrate (ThermoFisher; 34577) for visualization.

### TEER measurement

On day 8, purified BECs were seeded onto ECM coated 12 well transwell (Corning; 3460). TEER was measured using a EVOM II instrument (World Precisions). For an 8-day duration, the basal and apical layers were probed for TEER and received changes in EC- - media. A media-only transwell was used for temperature adjustment before the probe handled any measurements. TEER was additionally measured on three independent passages of CS79-hiPSCs for a period of 5 days post expansion.

### Dextran uptake assay

On day 8, purified CS79-derived BECs were seeded onto ECM coated 24 well plates at 500,000 cells/cm^2^. CS79-hiPSCs were seeded onto the same plate at the same density. On day 10, cells were treated with 10 kD fluorescein dextran at 1 mg/mL (ThermoFisher; D1821). Plates were either incubated at 37°C + 5% CO_2_ or at 4°C for 30 min. Cells were then removed from incubation and lysed using 500 μL RIPA buffer per well. Fluorescence values were measured using a plate reader (Molecular Devices SpectramaxiD3). A BCA assay was then performed, and OD values were used for normalization.

### Sodium fluorescein transport

We performed sodium fluorescein permeability assays as previously described (Stebbins et al., 2016). Briefly, we resuspend sodium fluorescein (Sigma; F6377-100G) to a stock concentration of 10 mM in PBS and stored it at 4°C protected from light. To make a working concentration, we diluted the stock solution in EC medium for a final concentration of 10 μM. On day 8, cells were seeded at a density of 1 million cells/cm^2^ onto ECM coated transwells. A blank ECM coated transwell was included to use in later calculations. On day 10, TEER was measured. Once TEER was measured, the medium was removed and replaced with pre-warmed EC medium by adding 500 μL to the top chamber and 1,500 μL to the bottom chamber. The plate was then incubated at 37°C for 60 min. After incubation, TEER was measured again to determine initial barrier integrity. After, medium was aspirated in the top of the transwell and replaced with 500 μL of the 10 μM working concentration of sodium fluorescein in blank and seeded transwells. After 15 min, 150 μL was collected from the bottom chamber of each transwell and placed into a 96-well plate. The removed medium was replaced with 150 μL of pre-warmed EC medium. This process was repeated every 15 min at 30, 45, and 60 min. At 60 min, an additional 150 μL from the top of the transwells was collected and transferred to a 96-well plate in addition to 150 μL of EC medium without sodium fluorescein. The solution from the top chambers were diluted to combat oversaturated signal. The fluorescence values were read using a plate reader (Molecular Devices SpectramaxiD3) at 485 nm excitation and 530 nm emission. We then calculated corrected signal, clearance volumes, the linear slope of clearance volume versus time, and the sodium fluorescein permeability using previously described formulas (Stebbins et al., 2016).

### Statistical analysis

Statistical analysis was performed using GraphPad Prism version 9.1.2 unless otherwise stated. Student’s *t*-test was used to determine significance for pair-wise comparison. Statistical significance was determined for a *p*-value of 0.05 or less. Error bars represent standard deviation (SD).

### Data availability

All data generated or analyzed during this study are included in this published article. hiPSC line CS79iBRCA-n2 available from Cedars-Sinai Biomanufacturing Center iPSC Core Repository.

## Results

### CS79iBRCA-n2 derived BECs possess endothelial properties

**FIGURE 1:**
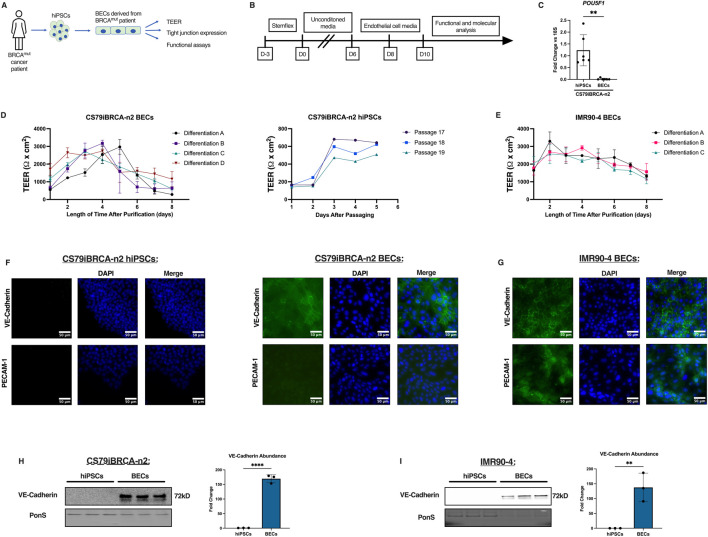
CS79iBRCA-n2-derived BECs have endothelial properties. **(A)** Graphic outlining the process of harvesting the hiPSCs and their differentiation into BECs. **(B)** Timeline outlining the differentiation process. **(C)** qPCR showing expression of pluripotency marker, *POUF51*, in CS79-hiPSCs compared to CS79-derived BECs (*N* = 6). **(D)** TEER measurements from CS79-derived BECs over a period of 8 days post purification from four independent differentiations conducted in triplicate per differentiation (*n* = 12). TEER measurements of three CS79-hiPSCs passages over a period of 5 days post passaging. **(E)** TEER measurements from IMR90-4 derived BECs over a period of 8 days post purification for three independent differentiations (*n* = 9). **(F)** Representative immunofluorescence images of CS79-hiPSCs and BECs for endothelial markers VE-Cadherin and PECAM-1 (green) with nuclei stained with DAPI (blue). **(G)** Representative immunofluorescence images of IMR90-4 derived BECs for endothelial markers VE-Cadherin and PECAM-1 (green) with nuclei stained with DAPI (blue). **(H)** Western blot analysis of CS79-hiPSCs and CS79-derived BECs probing for endothelial marker VE-Cadherin with densitometry quantification. PonS stain shown to show relative protein concentrations loaded for each lane (*n* = 3). **(I)** Western blot analyses of IMR90-4 hiPSCs and IMR90-4 derived BECs probing for endothelial marker VE-Cadherin with densitometry quantification. PonS stain shown to show relative protein concentrations loaded for each lane (*n* = 3). Scale bar = 50 μm. Statistical significance calculated by Student’s *t*-test, **p < 0.01, ****p < 0.0001. Error bars represent SD.

The CS79-hiPSCs were harvested from a *BRCA1* mutated invasive cancer patient (Figure 1A) and were then differentiated over the course of 13 days into BECs (Figure 1B). Using a previously published differentiation protocol, we were able to differentiate the CS79-hiPSCs into CS79-derived BECs by seeding the CS79-hiPSCs first onto Matrigel-coated flasks (Espinal et al., 2022; Stebbins et al., 2016). The CS79-iPSCs were then grown in unconditioned medium (UM) and differentiated to an endothelial cell and neural progenitor cell culture mixture (Lippmann et al., 2012; Lippmann et al., 2014). The switch to endothelial cell (EC) medium allows endothelial cells to be selectively expanded (Lippmann et al., 2014). On day 8, the BECs were purified onto extracellular matrix (ECM) coated plates (collagen IV, fibronectin, molecular water) which resulted in a purified monolayer of BECs (Figure 1B). We performed qPCR on CS79iBRCA-n2 hiPSCs and CS79-derived BECs to look at pluripotency marker Oct4 (*POU5F1*) where we saw a decrease of expression after differentiation (Figure 1C). To determine the barrier properties of the differentiated BECs, trans-endothelial electrical resistance (TEER) readings were obtained beginning on day 8 of the differentiation, when the BECs were seeded onto ECM coated plates (Stebbins et al., 2016; Sun et al., 2022). TEER was measured daily for 8 days after purification. This was repeated for four independent differentiations of CS79-derived BECs and results were similar throughout where TEER remained high until 5 days post purification when TEER began to decrease (Figure 1D). TEER was also recorded for three independent passages of CS79iBRCA-n2 hiPSCs to establish a baseline value (Figure 1D). Additionally, for a control cell line we used a well characterized hiPSC cell line, IMR90-4, and repeated the differentiation protocol. We then recorded TEER of three independent differentiations for 8 days post purification (Figure 1E).

Immunofluorescence images of CS79iBRCA-n2 hiPSCs were obtained for endothelial markers, vascular endothelial-cadherin (VE-Cadherin) and platelet endothelial cell adhesion molecule (PECAM-1) and showed no presence of endothelial markers in the hiPSCs (Figure 1F). However, the differentiated CS79-derived BECs displayed the localization of VE-Cadherin, whereas PECAM-1 showed limited expression and was not localized in the CS79-derived BECs (Figure 1F). Immunofluorescence images of the IMR90-4 BECs showed expression and localization of both VE-Cadherin and PECAM-1 (Figure 1G). To further confirm endothelial properties, Western blot analyses were performed for VE-Cadherin. Signal was observed in the CS79-derived BECs at 72 kDa corresponding to VE-Cadherin and no signal was observed for CS79-hiPSCs (Figure 1H), which was similar to the immunofluorescent staining images. We also performed Western blot analyses for VE-Cadherin on IMR90-4 hiPSCs and BECs, and observed signal in the IMR90-4 BECs and not in the IMR90-4 hiPSCs (Figure 1I). Together these data demonstrate that CS79iBRCA-n2 derived brain-like endothelial cells possess barrier function and some endothelial characteristics.

### Tight junction expression of CS79iBRCA-n2 derived BECs

**FIGURE 2:**
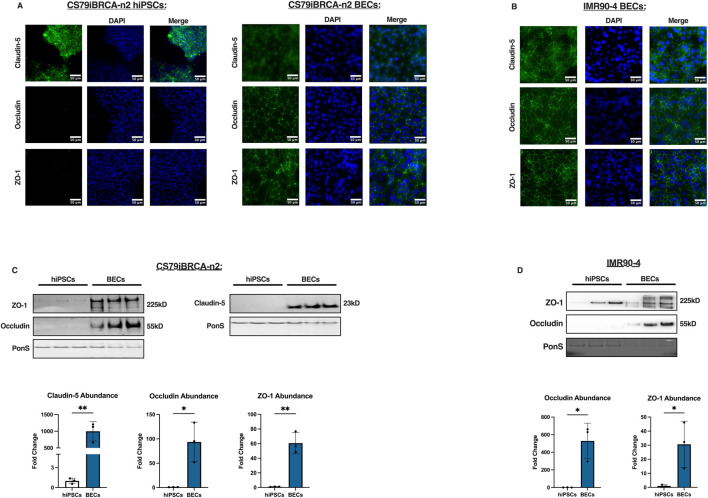
Tight junction expression of CS79iBRCA-n2-derived BECs. **(A)** Representative immunofluorescence images of CS79-hiPSCs and CS79-derived BECs for the tight junction proteins, ZO-1, Occludin, and Claudin-5 (green) and nuclei stained with DAPI (blue). Scale bar = 50 μm. **(B)** Representative immunofluorescence images of IMR90-4 derived BECs for tight junction proteins Claudin-5, Occludin, and ZO-1. Scale bar = 50 μm. **(C)** Western blot analyses of CS79-hiPSCs and CS79-derived BECs probing for tight junction proteins, ZO-1, Occludin, and Claudin-5. PonS stain shown to show relative protein concentrations loaded for each lane (*n* = 3). Quantification of western blots for ZO-1, Occludin, and Claudin-5 (*n* = 3). **(D)** Western blot analyses of IMR90-4 hiPSCs and IMR90-4 derived BECs probing for ZO-1 and Occludin. PonS stain shown to show relative protein concentrations loaded for each lane (*n* = 3). Quantification of western blots for ZO-1, Occludin, and Claudin-5 (*n* = 3). Statistical significance calculated by Student’s *t*-test, *p < 0.05, **p < 0.01. Error bars represent SD.

While hiPSCs produce tight junctions, they remain an important aspect of BBB function (Sun et al., 2022; Gastfriend et al., 2018). To characterize how the CS79-hiPSCs and the differentiated CS79-derived BECs expressed tight junction proteins, we measured the expression and localization of Zona Occludens-1 (ZO-1), Occludin, and Claudin-5 (Sun et al., 2021). Immunofluorescence images of the hiPSCs were obtained for ZO-1, Occludin, and Claudin-5. The hiPSCs expressed a high signal intensity for Claudin-5, which should be taken into consideration when utilizing this cell line (Figure 2A). The staining images revealed low signal intensity in the Occludin and ZO-1 tight junction proteins, indicating a low presence in CS79-hiPSCs (Figure 2A). However, when the same markers were measured in the differentiated CS79-derived BECs, we observed an increase in signal intensity in Occludin and ZO-1, suggesting the strong expression and localization of the tight junction proteins (Figure 2A). Additionally, we performed immunostaining on IMR90-4 BECs to show expression and localization of Claudin-5, Occludin, and ZO-1 (Figure 2B). Western blot analyses were conducted on CS79-hiPSCs and BECs using specific antibodies to confirm the presence of the tight junction proteins, ZO-1, Occludin, and Claudin-5. We obtained signal in the BEC samples at 225 kDa (corresponding to ZO-1), 55 kDa (corresponding to Occludin), and 23 kDa (corresponding to Claudin-5) respectively (Figure 2C).

Results demonstrate that there was a significantly higher abundance as seen by Western blotting of the tight junction proteins, ZO-1, Occludin, and Claudin-5, in the CS79-derived BECs than the hiPSCs (Figure 2C). We additionally performed Western blotting on IMR90-4 hiPSCs and BECs to observe expression of ZO-1 and Occludin which can be looked at as a reference of expression after BEC differentiation (Figure 2D). Together, these data suggest that the BEC differentiation increases the expression of tight junction proteins that contribute to barrier function.

### CS79iBRCA-n2 derived BECs express and localize transporter proteins

**FIGURE 3:**
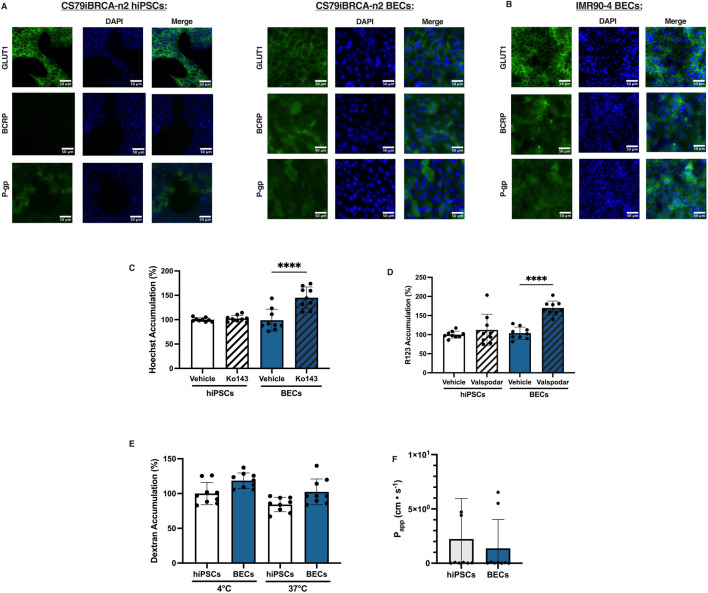
Characterization of the expression and localization of transporter proteins in CS79iBRCA-n2-derived BECs. **(A)** Representative immunofluorescence images for CS79-hiPSCs and CS79-derived BECs for nutrient transporter GLUT1, efflux transporter BCRP, and efflux transporter P-gp (green) and nuclei stained with DAPI (blue). Scale bar = 50 μm. **(B)** Representative immunofluorescence images for IMR90-4 derived BECs expression of GLUT1, BCRP, and P-gp (green) and nuclei staining with DAPI (blue). Scale bar = 50 μm. Substrate accumulation assays determining the function of the efflux transporters **(C)** BCRP and **(D)** P-gp for three independent differentiations conducted in triplicate (*n* = 9). **(E)** Uptake assay determining large molecule transport of 10 kD Dextran in CS79-hiPSCs and CS79-derived BECs (*n* = 9). **(F)** Sodium fluorescein transport assay in CS79-hiPSCs and CS79-derived BECs (*n* = 9). Student’s *t*-test was used to determine statistical significance within the same cell conditions, ****p < 0.0001. Error bars represent SD.

Next, we sought to observe other BBB properties such as the expression of nutrient transporters and multi-drug efflux transporters (Lippmann et al., 2012). Immunofluorescence images revealed that the nutrient transporter GLUT1 and efflux transporter P-gp was expressed and localized in the CS79-hiPSCs (Figure 3A). Expression of efflux transporter Breast Cancer Resistance Protein (BCRP) was not observed in the CS79-hiPSCs (Figure 3A). Immunofluorescence images of the CS79-derived BECs confirmed the expression and localization of nutrient transporter GLUT1, and the efflux transporters, P-gp and BCRP (Figure 3A). Additionally, expression and localization of GLUT1, BCRP, and P-gp was observed in IMR90-4-derived BECs was observed (Figure 3B). These data suggest that the BEC differentiation process increases some BBB-like transporter expression following hiPSC differentiation.

### CS79iBRCA-n2 derived BECs have a functional BCRP and P-gp efflux transport

To determine whether efflux transporters are functional in the CS79-derived BECs, we utilized and adjusted previously published substrate accumulation assay techniques to quantify P-gp and BCRP function (Stebbins et al., 2016; Kim et al., 2019; Engdahl et al., 2021). When the CS79-derived BECs were treated with the inhibitor, Ko143, we observed a significant increase in the accumulation of the BCRP substrate, Hoechst 33342, within the BECs. Hoechst 33342 accumulation within the cell increased by +50%, demonstrating that BCRP is functional in the BECs (Figure 3C) (Kim et al., 2019; Allen et al., 2002; Paturi et al., 2010). There was no significant increase in Hoechst 33342 accumulation within the CS79-hiPSCs when they were treated with Ko143 (Figure 3C). When the CS79-derived BECs were treated with the P-gp inhibitor, Valspodar, there was a significant increase in the accumulation of the P-gp substrate, Rhodamine 123 (R123). Valspodar treatment resulted in a +50% increase in R123 accumulation in the CS79-derived BECs, demonstrating functional P-gp is present in the CS79-derived BECs (Figure 3D). When CS79-hiPSCs were treated with Valspodar, there was no significant increase in substrate accumulation (Figure 3D) (Mayer et al., 1997). To further examine functional barrier integrity, we performed a large molecule transport assay using 10 kD FITC-Dextran (Figure 3E). We did not see a significant difference in accumulation between CS79-hiPSCs and CS79-derived BECs.

Additionally, we tested small molecule transport using sodium fluorescein where we saw a decrease of small molecule transport in CS79-derived BECs compared to CS79-hiPSCs (Figure 3F). Taken together we find that our differentiation process drives BBB-like phenotypes with the expression of functional efflux transporters BCRP and P-gp when compared to hiPSCs, but may not have robust transport ability of large molecules.

## Discussion

Brain metastasis is more prevalent in patients with *BRCA1* mutations (Ratner et al., 2019; Ben-Zion Berliner et al., 2024). Unfortunately, once brain metastasis occurs it is no longer considered curable (Narkhede et al., 2017). *In vitro* iPSC models used to study genetic mutations are limited due to the loss of patient specific genetic characteristics, as well as limited to number of passages before losing key phenotypic properties (Curry et al., 2015). To represent *BRCA1* mutations *in vitro*, a patient derived hiPSC line, CS79iBRCA-n2, was used that maintained the *BRCA1* disease mutation and patient’s genetic background. In this study we utilized a previously described differentiation protocol described to characterize the CS79iBRCA-n2 cell line after differentiation into BECs (Lippmann et al., 2012; Lippmann et al., 2014; Gomes et al., 2019). As a control, we utilized a well-characterized hiPSC line, IMR90-4, which has been extensively used to model the BBB, as well has been used with the differentiation protocol utilized in this study (Lippmann et al., 2012; Espinal et al., 2022; Hollmann et al., 2017; Stebbins et al., 2019).

After differentiation, TEER data across four independent differentiations showed tight monolayers with strong barrier integrity. The CS79iBRCA-n2 cell line has elevated TEER values consistent with other hiPSC lines used to generate BECs (Vatine et al., 2017; Lim et al., 2017; Aragón-González et al., 2024). CS79iBRCA-n2 hiPSCs were immunostained for endothelial specific markers VE-Cadherin and PECAM-1. No expression was visualized as expected. CS79iBRCA-n2-derived BECs were also stained for VE-Cadherin and PECAM-1. VE-Cadherin was well localized and distributed in the CS79-derived BECs. Additionally, VE-Cadherin protein abundance was visualized in CS79-derived BECs and not in CS79-hiPSCs which is consistent with the development of endothelial specific markers after differentiation.

To examine tight junction expression, we performed western blots and immunostaining on CS79iBRCA-n2 hiPSCs and CS79iBRCA-n2-derived BECs. We observed ZO-1, Occludin, and Claudin-5 expression and organization in the BECs which contributes to the selective permeability and barrier integrity of the BBB (Figure 2A). We also saw an increase of ZO-1, Occludin, and Claudin-5 protein abundance after differentiation in CS79-derived BECs (Figure 2C) (Urich et al., 2012). In a human immortalized endothelial cell line, CMEC/D3, low expression of Claudin-5 has been observed (Helms et al., 2016). In a different patient derived BBB model using the cell line CTR54F, low immunofluorescence intensity was observed for Claudin-5 and Occludin (Patel et al., 2017). For tight junction expression, the CS79iBRCA-n2 cell line demonstrates strong localization after BEC differentiation and can be used for relevant studies.

Additionally, we show through immunostaining that the CS79iBRCA-n2 derived BECs expressed drug efflux transporters P-gp and BCRP (Figure 3). To determine functionality of BCRP and P-gp we performed substrate accumulation assays, where functionality was observed in the CS79iBRCA-n2-derived BECs for both P-gp and BCRP. Other patient derived BBB models have seen a non-functional P-gp phenotype (Ozgür et al., 2023). Additionally, primary rodent BEC models have shown a downregulation of key BBB proteins such as GLUT-1 and P-gp (Helms et al., 2016). Interestingly, BCRP and P-gp expression was seen localized in the CS79iBRCA-n2-derived BECs. In addition, to investigate large molecule transport in the CS79-derived BECs and CS79-hiPSCs, we utilized dextran uptake assays. Though no significant difference was observed in large molecule accumulation, this could be contributed to the hiPSCs being grown to a full monolayer. Once grown to 100% confluence, the hiPSCs display some barrier properties, as shown by TEER values of the CS79-hiPSCs (Figure 1D). This could contribute to the lack of difference seen between accumulation. For future applications, it could be useful to be tested against a different barrier forming cell. To investigate small molecule transport, the sodium fluorescein assay displayed a decrease in small molecule transport in the CS79-derived BECs compared to the CS79-hiPSCs. Previous studies characterizing other hiPSC cell lines have demonstrated variation in BBB properties among differing cell lines (Vatine et al., 2017; Ozgür et al., 2023; Lim et al., 2017; Aragón-González et al., 2024). Similarly, the CS79-derived BECs express robust BBB phenotypes such as functional BCRP and P-gp and a trending decrease in small molecule transport. Based on these phenotypes, CS79iBRCA-n2 cell line can prove to be useful when studying P-gp or BCRP efflux transporter function. Further studies are needed to explore the functionality of GLUT1 and to what extent paracellular and transcellular transport is functional in the CS79-derived BECs.

Others have suggested that hiPSC-BECs in this protocol may not express VE-Cadherin expression and may represent an intermediate phenotype between neuroepithelium and brain endothelium (Lu et al., 2021). Here we demonstrate robust VE-Cadherin expression following this differentiation protocol suggesting an endothelial identity, however PECAM-1 expression was less convincing. Future work could expand on the differentiation by the inclusion of ETS transcription factors or small molecule cocktails that have been demonstrated to increase endothelial identity in this differentiation model (Porkoláb et al., 2023).

Overall, the CS79iBRCA-n2 derived BECs recapitulate some BBB-like properties such as high TEER, expression of tight junctions Claudin-5, ZO-1, and Occludin, and functional efflux transporters BCRP and P-gp.

## Data Availability

The raw data supporting the conclusions of this article will be made available by the authors, without undue reservation.

## References

[B1] Abbott N. J. Patabendige A. A. K. Dolman D. E. M. Yusof S. R. Begley D. J. (2010). Structure and function of the blood-brain barrier. Neurobiol. Dis. 37 (1), 13–25. 10.1016/j.nbd.2009.07.030 19664713

[B2] Allen J. D. van Loevezijn A. Lakhai J. M. van der Valk M. van Tellingen O. Reid G. (2002). Potent and specific inhibition of the breast cancer resistance protein multidrug transporter *in vitro* and in mouse intestine by a novel analogue of fumitremorgin C. Mol. Cancer Ther. 1 (6), 417–425. 12477054

[B3] Andjelkovic A. V. Stamatovic S. M. Phillips C. M. Martinez-Revollar G. Keep R. F. (2020). Modeling blood–brain barrier pathology in cerebrovascular disease *in vitro*: current and future paradigms. Fluids Barriers CNS 17 (1), 44. 10.1186/s12987-020-00202-7 32677965 PMC7367394

[B4] Aragón-González A. Shaw A. C. Kok J. R. Roussel F. S. Santos Souza C. D. Granger S. M. (2024). C9ORF72 patient-derived endothelial cells drive blood-brain barrier disruption and contribute to neurotoxicity. Fluids Barriers CNS 21 (1), 34. 10.1186/s12987-024-00528-6 38605366 PMC11007886

[B5] Arshad F. Wang L. Sy C. Avraham S. Avraham H. K. (2011). Blood-brain barrier integrity and breast cancer metastasis to the brain. Pathology Res. Int. 2011, 920509–920512. 10.4061/2011/920509 PMC3021881 21253507

[B6] Ben-Zion Berliner M. Yust-Katz S. Lavie I. Goldberg Y. Kedar I. Yerushalmi R. (2024). Central nervous system metastases in breast cancer patients with germline BRCA pathogenic variants compared to non-carriers: a matched-pair analysis. BMC Cancer 24 (1), 219. 10.1186/s12885-024-11975-7 38365640 PMC10870547

[B7] Boulton S. J. (2006). Cellular functions of the BRCA tumour-suppressor proteins. Biochem. Soc. Trans. 34 (5), 633–645. 10.1042/BST0340633 17052168

[B8] Curry E. L. Moad M. Robson C. N. Heer R. (2015). Using induced pluripotent stem cells as a tool for modelling carcinogenesis. World J. Stem Cells 7 (2), 461–469. 10.4252/wjsc.v7.i2.461 25815129 PMC4369501

[B9] Engdahl E. Van Schijndel M. D. M. Voulgaris D. Di Criscio M. Ramsbottom K. A. Rigden D. J. (2021). Bisphenol A Inhibits the transporter function of the blood-brain barrier by directly interacting with the ABC transporter breast cancer resistance protein (BCRP). Int. J. Mol. Sci. 22 (11), 5534. 10.3390/ijms22115534 34073890 PMC8197233

[B10] Espinal E. R. Sharp S. J. Kim B. J. (2022). Induced pluripotent stem cell (iPSC)-Derived endothelial cells to study bacterial-brain endothelial cell interactions. Methods Mol. Biol. 2492, 73–101. 10.1007/978-1-0716-2289-6_4 35733039

[B11] Gastfriend B. D. Palecek S. P. Shusta E. V. (2018). Modeling the blood-brain barrier: beyond the endothelial cells. Curr. Opin. Biomed. Eng. 5, 6–12. 10.1016/j.cobme.2017.11.002 29915815 PMC6003712

[B12] Gomes S. F. M. Westermann A. J. Sauerwein T. Hertlein T. Forstner K. U. Ohlsen K. (2019). Induced pluripotent stem cell-derived brain endothelial cells as a cellular model to study Neisseria meningitidis infection. Front. Microbiol. 10, 1181. 10.3389/fmicb.2019.01181 31191497 PMC6548865

[B13] Gudmundsdottir K. Ashworth A. (2006). The roles of BRCA1 and BRCA2 and associated proteins in the maintenance of genomic stability. Oncogene 25 (43), 5864–5874. 10.1038/sj.onc.1209874 16998501

[B14] He Y. Yao Y. Tsirka S. E. Cao Y. (2014). Cell-culture models of the blood–brain barrier. Stroke 45 (8), 2514–2526. 10.1161/STROKEAHA.114.005427 24938839 PMC4668829

[B15] Helms H. C. Abbott N. J. Burek M. Cecchelli R. Couraud P. O. Deli M. A. (2016). *In vitro* models of the blood-brain barrier: an overview of commonly used brain endothelial cell culture models and guidelines for their use. J. Cereb. Blood Flow Metab. 36 (5), 862–890. 10.1177/0271678X16630991 26868179 PMC4853841

[B16] Hersh D. S. Wadajkar A S. Roberts N. B. Perez G. J. Connolly N. P. Frenkel V. (2016). Evolving drug delivery strategies to overcome the blood brain barrier. Curr. Pharm. Des. 22 (9), 1177–1193. 10.2174/1381612822666151221150733 26685681 PMC4900538

[B17] Hollmann E. K. Bailey A. K. Potharazu A. V. Neely M. D. Bowman A. B. Lippmann E. S. (2017). Accelerated differentiation of human induced pluripotent stem cells to blood–brain barrier endothelial cells. Fluids Barriers CNS 14 (1), 9. 10.1186/s12987-017-0059-0 28407791 PMC5390351

[B18] Kim B. J. McDonagh M. A. Deng L. Gastfriend B. D. Schubert-Unkmeir A. Doran K. S. (2019). Streptococcus agalactiae disrupts P-glycoprotein function in brain endothelial cells. Fluids Barriers CNS 16 (1), 26. 10.1186/s12987-019-0146-5 31434575 PMC6704684

[B19] Knopp R. C. Banks W. A. Erickson M. A. (2022). Physical associations of microglia and the vascular blood-brain barrier and their importance in development, health, and disease. Curr. Opin. Neurobiol. 77, 102648. 10.1016/j.conb.2022.102648 36347075

[B20] Lim R. G. Quan C. Reyes-Ortiz A. M. Lutz S. E. Kedaigle A. J. Gipson T. A. (2017). Huntington’s disease iPSC-derived brain microvascular endothelial cells reveal WNT-mediated angiogenic and blood-brain barrier deficits. Cell Rep. 19 (7), 1365–1377. 10.1016/j.celrep.2017.04.021 28514657 PMC5646270

[B21] Lippmann E. S. Al-Ahmad A. Azarin S. M. Palecek S. P. Shusta E. V. (2014). A retinoic acid-enhanced, multicellular human blood-brain barrier model derived from stem cell sources. Sci. Rep. 4 (1), 4160. 10.1038/srep04160 24561821 PMC3932448

[B22] Lippmann E. S. Azarin S. M. Kay J. E. Nessler R. A. Wilson H. K. Al-Ahmad A. (2012). Derivation of blood-brain barrier endothelial cells from human pluripotent stem cells. Nat. Biotechnol. 30 (8), 783–791. 10.1038/nbt.2247 22729031 PMC3467331

[B23] Lu T. M. Houghton S. Magdeldin T. Durán J. G. B. Minotti A. P. Snead A. (2021). Pluripotent stem cell-derived epithelium misidentified as brain microvascular endothelium requires ETS factors to acquire vascular fate. Proc. Natl. Acad. Sci. U.S.A. 118 (8), e2016950118. 10.1073/pnas.2016950118 33542154 PMC7923590

[B24] Mai P. L. Chatterjee N. Hartge P. Tucker M. Brody L. Struewing J. P. (2009). Potential excess mortality in BRCA1/2 mutation carriers beyond breast, ovarian, prostate, and pancreatic cancers, and melanoma. PLoS One 4 (3), e4812. 10.1371/journal.pone.0004812 19277124 PMC2652075

[B25] Mayer U. Wagenaar E. Dorobek B. Beijnen J. H. Borst P. Schinkel A. H. (1997). Full blockade of intestinal P-glycoprotein and extensive inhibition of blood-brain barrier P-glycoprotein by oral treatment of mice with PSC833. J. Clin. Investig. 100 (10), 2430–2436. 10.1172/JCI119784 9366556 PMC508442

[B26] Nagai H. Kim Y. H. (2017). Cancer prevention from the perspective of global cancer burden patterns. J. Thorac. Dis. 9 (3), 448–451. 10.21037/jtd.2017.02.75 28449441 PMC5394024

[B27] Narkhede A. A. Shevde L. A. Rao S. S. (2017). Biomimetic strategies to recapitulate organ specific microenvironments for studying breast cancer metastasis. Int. J. Cancer 141 (6), 1091–1109. 10.1002/ijc.30748 28439901

[B28] Obermeier B. Daneman R. Ransohoff R. M. (2013). Development, maintenance and disruption of the blood-brain barrier. Nat. Med. 19 (12), 1584–1596. 10.1038/nm.3407 24309662 PMC4080800

[B29] Ozgür B. Puris E. Brachner A. Appelt-Menzel A. Oerter S. Balzer V. (2023). Characterization of an iPSC-based barrier model for blood-brain barrier investigations using the SBAD0201 stem cell line. Fluids Barriers CNS 20 (1), 96. 10.1186/s12987-023-00501-9 38115090 PMC10731806

[B30] Pardridge W. M. (2007). Blood–brain barrier delivery. Drug Discov. Today 12 (1-2), 54–61. 10.1016/j.drudis.2006.10.013 17198973

[B31] Patabendige A. Janigro D. (2023). The role of the blood–brain barrier during neurological disease and infection. Biochem. Soc. Trans. 51 (2), 613–626. 10.1042/BST20220830 36929707 PMC10212550

[B32] Patel R. Page S. Al-Ahmad A. J. (2017). Isogenic blood-brain barrier models based on patient-derived stem cells display inter-individual differences in cell maturation and functionality. J. Neurochem. 142 (1), 74–88. 10.1111/jnc.14040 28397247

[B33] Paturi D. K. Kwatra D. Ananthula H. K. Pal D. Mitra A. K. (2010). Identification and functional characterization of breast cancer resistance protein in human bronchial epithelial cells (Calu-3). Int. J. Pharm. 384 (1-2), 32–38. 10.1016/j.ijpharm.2009.09.037 19782742 PMC2830792

[B34] Porkoláb G. Mészáros M. Szecskó A. Vigh J. P. Walter F. R. Figueiredo R. (2023). Synergistic induction of blood-brain barrier properties. Proc. Natl. Acad. Sci. U.S.A. 121, e2316006121. 10.1073/pnas.2316006121 PMC11126970 38748577

[B35] Ratner E. Bala M. Louie-Gao M. Aydin E. Hazard S. Brastianos P. K. (2019). Increased risk of brain metastases in ovarian cancer patients with BRCA mutations. Gynecol. Oncol. 153 (3), 568–573. 10.1016/j.ygyno.2019.03.004 30876674

[B36] Rubin L. L. Staddon J. M. (1999). The cell biology of the blood-brain barrier. Annu. Rev. Neurosci. 22 (1), 11–28. 10.1146/annurev.neuro.22.1.11 10202530

[B37] Siegel R. L. Giaquinto A. N. Jemal A. (2024). Cancer statistics, 2024. CA A Cancer J. Clin. 74 (1), 12–49. 10.3322/caac.21820 38230766

[B38] Stebbins M. J. Gastfriend B. D. Canfield S. G. Lee M. S. Richards D. Faubion M. G. (2019). Human pluripotent stem cell-derived brain pericyte-like cells induce blood-brain barrier properties. Sci. Adv. 5 (3), eaau7375. 10.1126/sciadv.aau7375 30891496 PMC6415958

[B39] Stebbins M. J. Wilson H. K. Canfield S. G. Qian T. Palecek S. P. Shusta E. V. (2016). Differentiation and characterization of human pluripotent stem cell-derived brain microvascular endothelial cells. Methods 101, 93–102. 10.1016/j.ymeth.2015.10.016 26518252 PMC4848177

[B40] Sun H. Hu H. Liu C. Sun N. Duan C. (2021). Methods used for the measurement of blood-brain barrier integrity. Metab. Brain Dis. 36 (5), 723–735. 10.1007/s11011-021-00694-8 33635479

[B41] Sun J. Ou W. Han D. Paganini-Hill A. Fisher M. J. Sumbria R. K. (2022). Comparative studies between the murine immortalized brain endothelial cell line (bEnd.3) and induced pluripotent stem cell-derived human brain endothelial cells for paracellular transport. PLoS One 17 (5), e0268860. 10.1371/journal.pone.0268860 35613139 PMC9132315

[B42] Urich E. Lazic S. E. Molnos J. Wells I. Freskgård P.-O. (2012). Transcriptional profiling of human brain endothelial cells reveals key properties crucial for predictive *in vitro* blood-brain barrier models. PLoS One 7 (5), e38149. 10.1371/journal.pone.0038149 22675443 PMC3364980

[B43] Vatine G. D. Al-Ahmad A. Barriga B. K. Svendsen S. Salim A. Garcia L. (2017). Modeling psychomotor retardation using iPSCs from MCT8-deficient patients indicates a prominent role for the blood-brain barrier. Cell Stem Cell 20 (6), 831–843. 10.1016/j.stem.2017.04.002 28526555 PMC6659720

[B44] Verscheijden L. F. M. Koenderink J. B. De Wildt S. N. Russel F. G. M. (2021). Differences in P-glycoprotein activity in human and rodent blood–brain barrier assessed by mechanistic modelling. Arch. Toxicol. 95 (9), 3015–3029. 10.1007/s00204-021-03115-y 34268580 PMC8380243

[B45] Wang H. Naghavi M. Allen C. Barber R. M. Bhutta Z. A. Carter A. (2016). Global, regional, and national life expectancy, all-cause mortality, and cause-specific mortality for 249 causes of death, 1980–2015: a systematic analysis for the Global Burden of Disease Study 2015. Lancet 388 (10053), 1459–1544. 10.1016/S0140-6736(16)31012-1 27733281 PMC5388903

[B46] Wilhelm I. Krizbai I. A. (2014). *In vitro* models of the blood–brain barrier for the study of drug delivery to the brain. Mol. Pharm. 11 (7), 1949–1963. 10.1021/mp500046f 24641309

[B47] Yucer N. Ahdoot R. Workman M. J. Laperle A. H. Recouvreux M. S. Kurowski K. (2021). Human iPSC-derived fallopian tube organoids with BRCA1 mutation recapitulate early-stage carcinogenesis. Cell Rep. 37 (13), 110146. 10.1016/j.celrep.2021.110146 34965417 PMC9000920

